# Disrupted Functional Network Topology in Children and Adolescents With Post-traumatic Stress Disorder

**DOI:** 10.3389/fnins.2018.00709

**Published:** 2018-10-09

**Authors:** Jian Xu, Fuqin Chen, Du Lei, Wang Zhan, Xiaomeng Sun, Xueling Suo, Zulai Peng, Ting Wang, Junran Zhang, Qiyong Gong

**Affiliations:** ^1^Department of Medical Information Engineering, School of Electrical Engineering and Information, Sichuan University, Chengdu, China; ^2^Department of Radiology, Huaxi MR Research Center, West China Hospital of Sichuan University, Chengdu, China; ^3^Neuroimaging Center, University of Maryland, Rockville, MD, United States; ^4^The General Hospital of Chinese People's Armed Police Force, Beijing, China; ^5^Chongqing Mental Health Center, Chongqing, China; ^6^Department of Computer Science, Chengdu University of Information Technology, Chengdu, China

**Keywords:** children and adolescents, PTSD, graph theory, topology, small-world

## Abstract

Neuroimaging studies in children and adolescents with post-traumatic stress disorder (PTSD) have focused on abnormal structures and the functionality of a few individual brain regions. However, little is known about alterations to the topological organization of whole-brain functional networks in children and adolescents with PTSD. To this end, we investigated the topological properties of brain functional networks derived from resting-state functional magnetic resonance imaging (r-fMRI) in patients suffering from PTSD. The r-fMRI data were obtained from 10 PTSD patients and 16 trauma-exposed non-PTSD subjects. Graph theory analysis was used to investigate the topological properties of the two groups, and group comparisons of topological metrics were performed using nonparametric permutation tests. Both the PTSD and non-PTSD groups showed the functional brain network to have a small-world architecture. However, the PTSD group exhibited alterations in global properties characterized by higher global efficiency, lower clustering coefficient, and characteristic path length, implying a shift toward randomization of the networks. The PTSD group also showed increased nodal centralities, predominately in the left middle frontal gyrus, caudate nucleus, and hippocampus, and decreased nodal centralities in the left anterior cingulate cortex, left paracentral lobule, and bilateral thalami. In addition, the clustering coefficient and nodal betweenness of the left paracentral lobule were found to be negatively and positively correlated with the re-experiencing and hyper-arousal symptoms of PTSD respectively. The findings of disrupted topological properties of functional brain networks may help to better understand the pathophysiological mechanism of PTSD in children and adolescents.

## Introduction

Post-traumatic stress disorder (PTSD), a common psychiatric disorder that develops after people have been exposed to catastrophic mental trauma, has been characterized with typical symptoms such as re-experiencing, hyper-arousal, and avoidance (Yehuda, 2002). The overall lifetime prevalence of PTSD in the youth population is 3–9% (Kilpatrick et al., 2003; Copeland et al., 2010). It is well documented that children and adolescents are more susceptible to developing PTSD after catastrophic events than are adults (Silva et al., 2000). Previous studies have suggested that the pathogenesis of PTSD in children and adolescents differs from that in adults, on account of neurodevelopment, psychological tolerance, and individual differences (Santosh, 2000; Casey et al., 2008; Crone, 2009).

The majority of studies on PTSD have been performed in the adult population, with only a few neuroimaging studies having focused on children and adolescents with PTSD (De Bellis et al., 2002b; Rinne-Albers et al., 2013). For the latter groups, the main findings reported are regional structural changes, such as those to the hippocampus (Tupler and De Bellis, 2006), corpus callosum (Jackowski et al., 2008), superior temporal gyrus (De Bellis et al., 2002a), and prefrontal cortex (Richert et al., 2006; Carrion et al., 2009). There are less neuroimaging studies that have examined functional alterations in young PTSD patients. Yang et al. found that adolescent PTSD patients demonstrated higher activation in the left parahippocampal gyrus, bilateral visual cortex, and cerebellum during earthquake imagery recall, and lower activation in the anterior cingulate gyrus during an earthquake perception state (Yang et al., 2004). Carrion et al. found that youths with post-traumatic stress symptoms exhibited frontal activity during response-inhibition tasks (Carrion et al., 2008).

The human brain is a complex network with an optimal balance between local specialization and global integration. Recent advances in graph theoretical analysis of resting-state functional magnetic imaging (r-fMRI) data have provided a powerful framework to characterize the topological properties of the brain functional network (Bullmore and Bassett, 2011; Sporns, 2011). This approach enables the characterization of the human brain as a large-scale network consisting of nodes (brain regions) and edges (connections between nodes), and has been applied to the study of various brain-related diseases, such as schizophrenia (Liu et al., 2008), major depressive disorder (Zhang et al., 2011), epilepsy (Xiao et al., 2015), and Alzheimer's disease (Sanz-Arigita et al., 2010). Several recent studies on PTSD have reported abnormalities in the small-world properties of brain networks derived from different modalities, including structural networks based on diffusion tensor imaging (DTI) tractography (Long et al., 2013), structural covariance networks based on gray matter volume/thickness (Mueller et al., 2015), and resting-state functional networks (Lei et al., 2015; Suo et al., 2015). However, few studies have focused on the whole-brain functional networks of children and adolescents with PTSD.

Here, we hypothesize that PTSD in children and adolescents disrupts the topological organization of intrinsic functional brain networks, and that these alterations may be related to the three typical symptoms (re-experiencing, hyper-arousal, and avoidance) of PTSD. To test our hypothesis, we constructed brain functional networks with r-fMRI data acquired from both PTSD patients and control subjects, and analyzed the topological organization of their brain networks using graph theory tools. Furthermore, we investigated correlations between the altered network metrics and clinical variables.

## Materials and methods

### Subjects

The current study was approved by the ethical committee of West China Hospital, and written informed consent was obtained from guardians of all participants. All subjects were children or adolescents, whose ages ranged from 8 to 17 years. They all were drawn from a large-scale PTSD survey of post-earthquake survivors in Sichuan province, China, which was hit by an 8.0-magnitude earthquake on May 12, 2008. They were carefully screened with the PTSD checklist (PCL). Those who scored >35 on the PCL were further screened with the Clinician-Administered PTSD Scale (CAPS), and those subjects with a CAPS score of >50 were eligible for further evaluation for inclusion in the PTSD group. The participants were interviewed by two experienced psychiatrists to confirm the PTSD diagnosis in the individuals who were suspected of having the disorder according to their CAPS scores, and to exclude any psychiatric co-morbidities using the structured clinical interview for DSM-IV Axis I disorders (SCID). The exclusion criteria for the PTSD group were: (1) current or past other psychiatric disorders; (2) IQ < 80; (3) use of psychotropic medications in the past 4 weeks; (4) any significant medical or neurological conditions or history of head injury; (5) left-handedness; and (6) a magnetic resonance imaging (MRI) contraindication.

The traumatized control subjects, whose ages ranged from 8 to 17 years-old, were evaluated using the SCID and scored below 35 on the CAPS. They likewise experienced the May 12 earthquake, but did not suffer from PTSD. The exclusion criteria for the controls were the same as for the PTSD group. Diagnostic-quality brain images were reported by two experienced neuroradiologists. The final study consisted of 10 right-handed PTSD patients and 16 right-handed traumatized control subjects. The detailed demographics and clinical characteristics of the participants are presented in Table 1.

**Table 1:** Demographics and clinical characteristics of the subjects^a^.

**Characteristics**	**Non-PTSD(*n* = 16)**	**PTSD(*n* = 10)**	***P-* value**
Age^b^ (year)	14.63 ± 1.59	14.40 ± 2.67	0.813^d^
Gender (male/female)	5/11	3/7	0.946^e^
Education^c^ (years)	8.63 ± 1.59	8.40 ± 2.67	0.813^d^
Course of diseaseb (months)	19.32 ± 0.07	19.29 ± 0.09	0.369^d^
Handedness (R/L)	16/0	10/0	–
CAPS (total)	6.94 ± 5.16	68.90 ± 16.13	<0.001
Reexperience	2.56 ± 1.63	17.10 ± 5.47	<0.001
Avoidance	2.13 ± 3.14	28.70 ± 9.82	<0.001
Hyper-arousal	2.25 ± 2.91	23.10 ± 7.80	<0.001

a *Data are presented as the mean ± SD*.

b *Age and course of disease were defined as the time of magnetic resonance scanning*.

c *Years of education refers to the total number of years of completed education, as reported by the participant*.

d *The P value was obtained by a two-sample two-tailed t-test*.

e *The P Value was obtained using a two-tailed Pearson chi-square test*.

*PTSD, post-traumatic stress disorder; CAPS, Clinician-Administered PTSD Scale*.

### Data acquisition and preprocessing

All subjects underwent an r-fMRI scan using a 3T magnetic resonance system (Siemens Medical Systems, Erlangen, Germany) with an 8 channel phased array head coil. The sequence parameters were as follows: repetition time/echo time (TR/TE) = 2,000/30 ms; slice thickness = 5 mm (no slice gap); flip angle = 90°; field of view (FOV) = 240 × 240 mm^2^; matrix size = 64 × 64; voxel size 3.75 × 3.75 × 5 mm^3^; interleaved scanning. Each brain volume consisted of 30 axial slices and each functional run contained 205 image volumes. Subjects were instructed to relax with their eyes closed for the duration of the scan, but to not fall asleep.

The r-fMRI data preprocessing was carried out using SPM8 (http://www.fil.ion.ucl.ac.uk/spm). The first 10 time points were discarded to avoid instability in the initial MRI signal. The remaining fMRI data were corrected for intravolume acquisition time delay and head motion. The head motion parameters of all participants were determined, and the inclusion criteria were extended to include translational movement <3 mm and rotation <3°. After these corrections, the images were spatially normalized to a standard space, the Montreal Neurologic Institute (MNI) 152 template resampled at a 3 × 3 × 3 mm resolution. The resulting normalized functional images were spatially smoothed (Gaussian kernel with a full width at half maximum of 4 mm) and were linearly detrended. The images were further temporally band-pass filtered (0.01–0.08 Hz) to remove low-frequency drift and high-frequency physiological noise. Finally, nuisance covariates, including the white matter signal, the cerebrospinal fluid signal, and the motion parameters (three translational and three rotational parameters), were regressed out.

### Network construction and analysis

The network was constructed using GRETNA (http://www.nitrc.org/projects/gretna/). To define the network nodes, the Automated Anatomic Labeling atlas (Tzourio-Mazoyer et al., 2002) was used to parcellate the whole brain into 90 cortical and subcortical regions of interest, with each region representing a node of the network. To define the network edges, the mean time series of each region was acquired and then used to compute the partial correlation, resulting in a 90 × 90 partial correlation matrix for each subject. Finally, the matrices were converted into binarized matrices (i.e., adjacency matrices) according to a predefined threshold. This process has been used in previous brain network studies (Zhang et al., 2011; Lei et al., 2015; Suo et al., 2015).

We applied a sparsity threshold S to all correlation matrices, which guaranteed that the thresholded networks had the same number of nodes and edges by applying a subject-specific correlation coefficient threshold, which minimized the effects of possible discrepancies in the overall correlation strength between groups. To ensure maintenance of small-worldness (scalar σ larger than 1.1) and have sparse properties with as few spurious edges as possible for the thresholded networks, our generated threshold range was 0.10 < S < 0.34, with an interval of 0.01.

**Figure 1:**
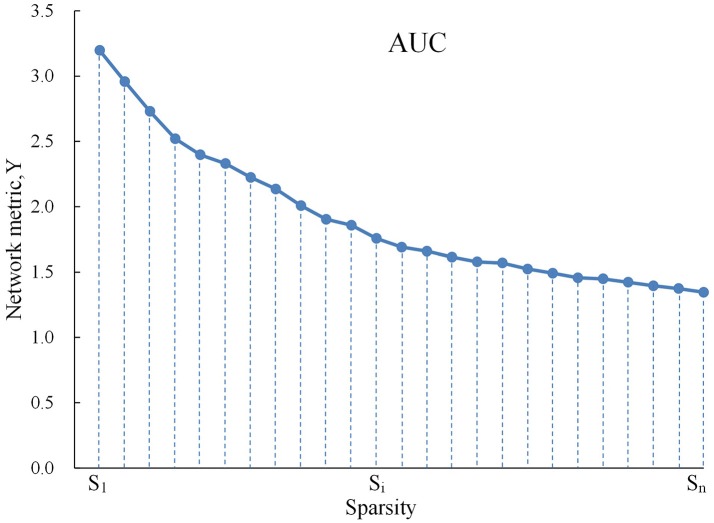
Graph shows AUC for a network metric Y, which was calculated over the sparsity thresh- old range of S1 to Sn with an interval of Δs as follows: $Y^{AUC}=\frac{1}{2}\overset{N-1}{\underset{k=1}{\sum }}\left[Y\left(S_{k}\right)+Y\left(S_{k+1}\right)\right]•\Delta S$. In the current study, S_1_ = 0.10, S_n_ = 0.34, and Δs = 0.01.

For brain networks at each sparsity threshold, we calculated both global and nodal metrics. The global metrics included (1) small-world parameters involving clustering coefficient Cp, characteristic path length Lp, normalized clustering coefficient γ, normalized characteristic path length λ, and small-worldness σ; (2) network efficiency parameters including the global efficiency Eglob and local efficiency Eloc. The regional measures included three nodal centrality metrics: the efficiency e, degree k, and betweenness b. We calculated the area under the curve (AUC) for each network metric, which provides a summarized scalar for the topological characterization of brain networks independent of a single threshold selection (Figure 1; Lei et al., 2015). The AUC metric has been used in previous brain network studies and is sensitive for the detection of topological alterations of brain disorders.

### Statistical analysis

Because AUC describes the topology attributes of brain network generally, and it can select single threshold calculation independently, moreover, it is highly sensitive about topology structure of brain disease abnormally. We calculated the AUC for each network metric, which provides a summarized scalar for the topological characterization of brain networks independent of a single threshold selection (Suo et al., 2015). The AUC for a general metric Y can be defined as the AUC for calculating the sparsity range from S_1_ to S_n_ (the interval is Δs), and it's formula can be described as Lei et al. (2015):

$$\begin{array}{l} Y^{AUC}=\frac{1}{2}\overset{N-1}{\underset{k=1}{\sum }}\left[Y\left(S_{k}\right)+Y\left(S_{k+1}\right)\right]•\Delta S \end{array}$$

To determine whether there are inter-group differences in network attributes, the non-parametric permutation test is used to test the AUC of each network attribute in the two sets of samples. It is divided into the following steps:

1. Building the null hypothesis. It is assumed that there is no difference between the means of the two statistical samples.
2. Determining inspection level α, which is designated as 0.05.
3. Calculating test statistic of previous two groups' samples.
4. To determine whether the differences between groups of network attributes could occur accidentally, a sample is selected at random without replacement from sample observation (two samples get together) so as to regroup. And then computing replacement statistical tests two samples ACU after stochastic grouping.
5. Setting the number of random packets, for example 10,000 times, repeat step 4 1,000 times. Then get a empirical exampling distribution of replacement statistical tests.
6. Adopting 95% of every empirical exampling distribution as critical value of null tail test of the null hypothesis. This kind of mistakes is kept within 0.05, and calculating odds is p.
7. Owing to inspection level (significance level) given by step 2, according to the principle of small probability, make the conclusion.

Furthermore, to address the problem of multiple comparisons, the nodal centralities were tested with regard to whether they survived a Benjamini-Hochberg false discovery rate (FDR) correction method at the expected significance level of 0.05.

After the significant between-group differences had been identified in the network metrics, we assessed the relationships between these altered metrics and the CAPS (total scores, and re-experiencing, avoidance, and hyper-arousal subscale scores) in the PTSD group, using partial correlations with age and gender as covariates.

The statistical analysis of the demographic and clinical data was conducted using SPSS software (http://www.spss.com), version 17.0 (Chicago, IL), and *P* < 0.05 was considered statistically significant.

## Results

### Demographics and clinical comparisons

Group comparisons are based on 10 patients (age 14.40 ± 2.67 years, range 8–17 years) and 16 traumatized control subjects (age 14.63 ± 1.59 years, range 10–17 years). The PTSD patients and non-PTSD controls showed no significant between-group differences in age, gender, education, and course of disease (*P* > 0.05, Table 1).

### Alterations in global network topology

**Figure 2:**
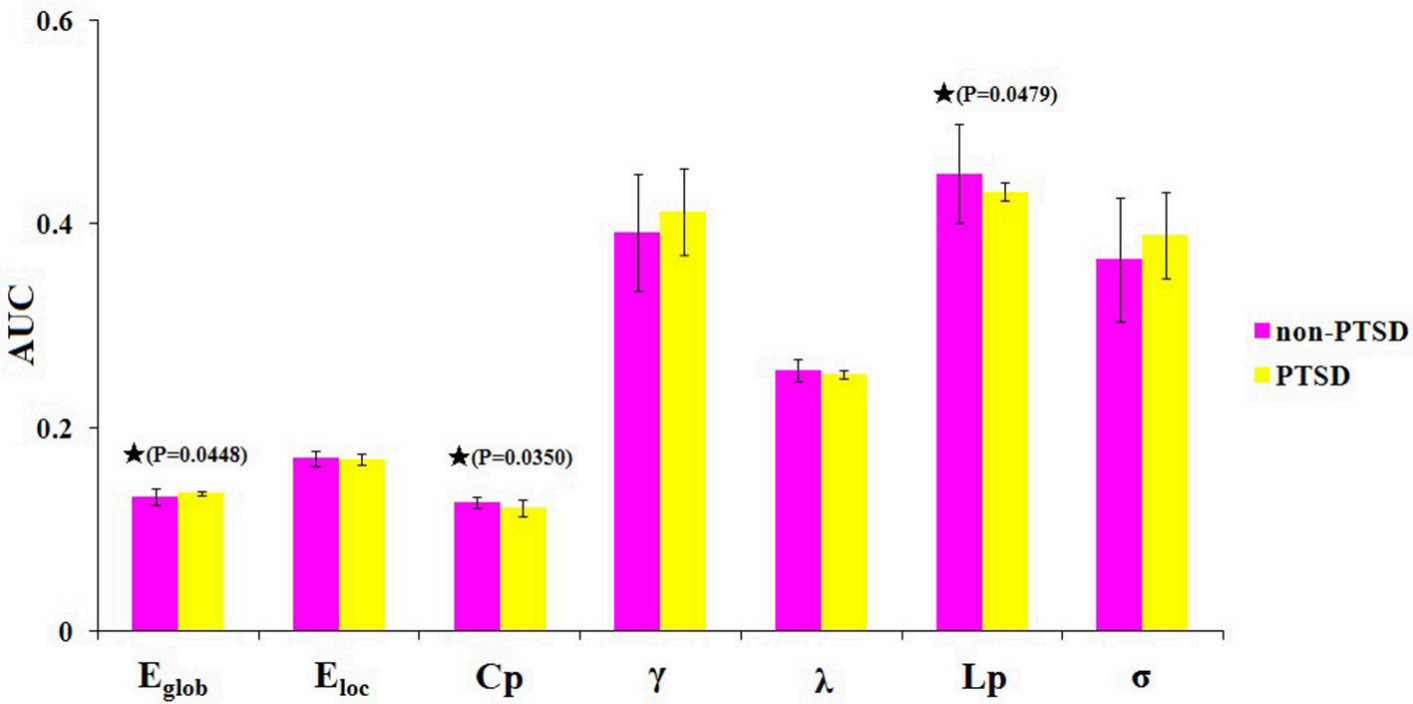
The differences in topological properties of functional brain networks between the PTSD and control groups. Significant differences were found in Eglob (*P* = 0.0448), Cp (*P* = 0.0350), and Lp (*P* = 0.0479) in children and adolescents with PTSD patients. The black stars indicate the significantly statistical difference between the two groups (nonparametric permutation test, *P* < 0.05, uncorrected), error bars denote standard deviations. Eglob, the global efficiency; Eloc, local efficiency; Cp, the clustering coefficient; γ, normalized clustering coefficient; λ, normalized characteristic path length; Lp, the characteristic path length; σ, small-worldness. AUC, area under the curve.

Within the defined threshold range (0.10–0.34), both children and adolescents in the PTSD and non-PTSD control groups exhibited small-world topology (γ > 1 and λ ≈ 1) in the brain functional connectome. Compared with the non-PTSD controls, the PTSD group showed significantly lower values in both the clustering coefficient Cp (*P* = 0.0350) and characteristic path length Lp (*P* = 0.0479). No significant differences were found in γ, λ, or σ. As for network efficiency, the PTSD group revealed a significantly increased global efficiency Eglob (*P* = 0.0448) but unchanged local efficiency Eloc (*P* = 0.4165) in the functional brain networks (Figure 2).

### Alterations in local network topology

**Figure 3:**
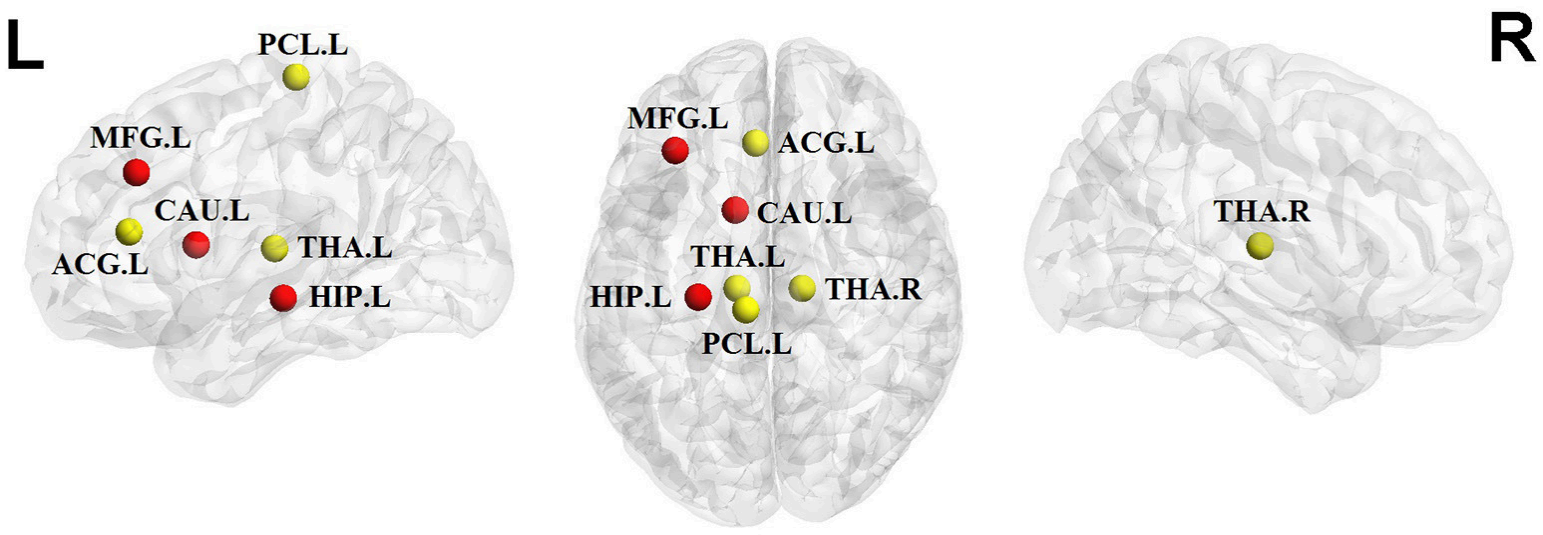
Significant alterations of nodal centralities in PTSD group compared to trauma-exposed non-PTSD group (*P* < 0.05, uncorrected). Nodes in red/yellow denote regions with increased/decreased nodal centralities in the patients. MFG, middle frontal gyrus; ACG, anterior cingulate gyrus; PCL, paracentral lobule; CAU, caudate nuleus; THA, thalamus; HIP, hippocampus; R, right hemisphere; P, posterior. The nodes were mapped onto the cortical surfaces using the BrainNet Viewer package (http://www.nitrc.org/ projects/bnv).

We identified brain regions showing significant between-group differences in at least one nodal metric (*P* < 0.05, uncorrected). Relative to the control group, the children and adolescents with PTSD exhibited increased nodal centralities in the left middle frontal gyrus, caudate nucleus, and hippocampus. Decreased nodal centralities were found in the left anterior cingulate cortex, left paracentral lobule, and bilateral thalami (Table 2, Figure 3).

**Table 2:** *P*-value for regions showing altered nodal centralities in children and adolescents with PTSD group vs. trauma-exposed non-PTSD control group.

**Brain region**	**Nodal betweeness**	**Nodal degree**	**Nodal efficiency**
**PTSD** > **NON-PTSD**			
Left middle frontal gyrus	**0.007899**	**0.027197**	**0.017398**
Left caudate nucleus	**0.023298**	0.376962	0.281272
Left hippocampus	0.125287	0.129187	**0.034197**
**PTSD < NON-PTSD**			
Left anterior cingulate gyrus	**0.044396**	0.357664	0.458054
Left paracentral lobule	**0.042796**	0.169683	0.483452
Left thalamus	**0.048795**	**0.033297**	0.059794
Right thalamus	0.142486	**0.042796**	0.070893

*Region were considered abnormal in the PTSD patients if they exhibited significant between-group differences (P < 0.05, uncorrected) in at least one of the three nodal centralities (shown in bold font). All P values were obtained by using a permutation test*.

### Relationships between altered network metrics and clinical variables

**Figure 4:**
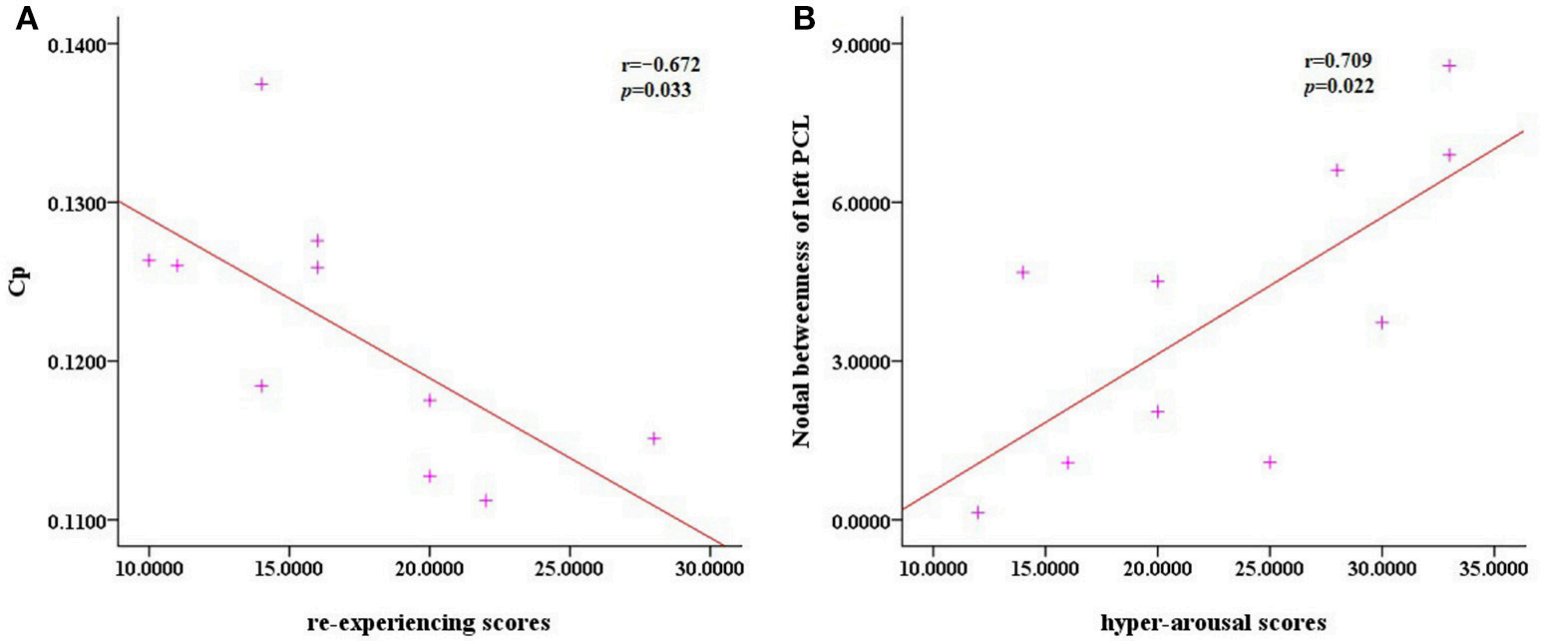
Scatter plots of Cp against the re-experencing subscale and the nodal betweenness of the left PCL compared to hyper-arousal subscale. **(A)** The correlation analysis indicated that in the PTSD patients, the clustering coefficient Cp was negatively correlated with the re-experencing subscale (*P* = 0.033). **(B)** The nodal betweenness of the left PCL was positively correlated with the hyper-arousal subscale (*P* = 0.022).

There were no significant correlations between the altered network metrics and the CAPS scores (*P* > 0.05). With regard to the subscale CAPS scores, the clustering coefficient was negatively correlated with the re-experiencing subscale (*P* = 0.033) and the left paracentral lobule was positively correlated with the hyper-arousal subscale (*P* = 0.022; Figure 4).

## Discussion

In this study using graph theory analysis, we constructed functional networks, analyzed the network topological properties, and investigated between-group differences and relationships with the subscale CAPS scores. Our main findings are summarized as follows: (1) At the global level, the PTSD group showed significant increases in global efficiency, and decreases in the clustering coefficient and characteristic path length, implying a disturbance of the normal global integration of whole-brain networks and a shift toward randomization of the networks. (2) At the nodal level, significantly increased nodal centralities were detected primarily in the left middle frontal gyrus, caudate nucleus, and hippocampus, but decreased nodal centralities were found in the left anterior cingulate cortex, left paracentral lobule, and bilateral thalami. (3) In the correlation analysis, the clustering coefficient and nodal betweenness of the left paracentral lobule were found to be negatively and positively correlated, respectively, with the re-experiencing and hyper-arousal symptoms of PTSD. We believe that these findings may contribute to a better understanding of the disrupted topological organization of whole-brain functional networks in children and adolescents with PTSD.

### Alterations in global network organization

The PTSD group exhibited significant increased global efficiency, and decreased clustering coefficient and characteristic path length, which may be ascribed to increased long-distance functional connections between remote brain areas, implying a disturbance to the normal global integration of whole-brain networks in PTSD patients. Consistent with a previous DTI tractography study (Long et al., 2013), our findings also showed enhanced global integration (decreased characteristic path length) and disrupted local segregation (decreased clustering coefficient) in the patients. Together with the fact that the clustering coefficient was negatively correlated with the re-experiencing symptom of PTSD, these findings suggest that functional brain networks in children and adolescents with PTSD are closer to a randomized configuration. This randomization process, in which the network transforms from a small-world to a random network, has been observed in other neuropsychiatric disorders such as schizophrenia (Liu et al., 2008), major depressive disorder (Zhang et al., 2011), and Alzheimer's disease (Sanz-Arigita et al., 2010). In general, higher global efficiency and lower characteristic path length indicate a greater ability to integrate information from the brain network. Therefore, these changes in specific global topological properties may reflect disrupted neuronal network organization in children and adolescents with PTSD.

### Alterations in local network metrics

In the PTSD patients, increases in the local metrics of betweenness, degree, and nodal efficiency were observed in the left middle frontal gyrus, caudate nucleus, and hippocampus, while reductions were mainly found in the left anterior cingulate cortex, left paracentral lobule, and bilateral thalami. The left middle frontal gyrus, which has been implicated in the salience network (Seeley et al., 2007), has often been associated with set-shifting processes in cognitive (Lie et al., 2006) and emotional tasks (Simmons et al., 2009). The caudate nucleus is a key brain structure involved in the regulation of cognition and mood (Lehéricy and Gerardin, 2002); only a few studies have reported smaller caudate nuclei in adults with a history of traumatic adverse childhood events (Cohen et al., 2006). Changes in the caudate nucleus may be a result of early stress and traumatic events on the developing brain. Therefore, the abnormalities in these two areas found in this study may reflect the outcome of disrupted cognitive and emotional mechanisms in PTSD. Moreover, dysfunction in the hippocampus may underlie PTSD-related deficits in learning and contextual memory (e.g., the inability to extinguish a fear response) (Acheson et al., 2012; Patel et al., 2012). The hippocampus has abundant glucocorticoid receptors, which are associated with the acute release of glucocorticoids via activation of the hypothalamic-pituitary-adrenal (HPA) axis after stress; it is thus linked with a negative feedback to shut off HPA axis activation (Sapolsky, 2003). Evidence from a psychobiological study revealed altered catecholamines and HPA axis activity in children and adolescents with maltreatment-related PTSD (De Bellis et al., 2002b). Based on these studies, we speculate that the observed PTSD-related network shift abnormalities might be associated with altered HPA axis activity caused by an aberrant hippocampus. Recent studies have suggested decreased local network connectivity of the ACC in PTSD patients (Cisler et al., 2014; Kennis et al., 2016), so decreased nodal centralities in the left ACC may therefore be an indication of the disruption of attention, emotional regulation, and conditioned fear processing in the pathology of PTSD (Patel et al., 2012). We also found decreased nodal centralities in the bilateral thalami, which along with the ACC are the components of the salience network (SN) responsible for detecting and responding to salient stimuli (Seeley et al., 2007). FMRI (Lanius et al., 2003), single photon emission computed tomography (SPECT) (Kim et al., 2007), and positron emission tomography (PET) (Neumeister and Sobin, 2012) studies have all found the thalamus to be involved in PTSD, against the background that it could be implicated in regulation of consciousness, sleep, alertness, and memory. Taken together, our findings of decreased nodal centralities of the two areas may be indicative of the disruptions in emotional and cognitive processing in PTSD patients. In addition, PTSD-related alterations in the nodal centralities in the left paracentral lobule were also found to be positively correlated with the hyper-arousal symptoms of PTSD, suggesting that abnormalities in the left paracentral lobule could be associated with the dyscoimesis of the hyper-arousal manifestation in PTSD patients (Shaw et al., 2002).

Several limitations of the current study deserve attention. Because of the small sample size, the nodal centrality results did not survive the application of corrections for multiple comparisons (an FDR correction), meaning that the statistical power is limited. Future investigation should be conducted using a larger sample of PTSD patients. For controls, we selected a population that was exposed to the earthquake without developing PTSD; further studies are desirable with an additional group of non-traumatized individuals to provide a more comprehensive insight into the functional networks of PTSD in children and adolescents (Kim et al., 2007).

## Conclusion

Our results showed disruptions in the functional network topology in children and adolescents with PTSD, implying a shift toward randomization in their brain networks. These alterations in the specific topological metrics may help provide a better understanding of the pathophysiological mechanisms of PTSD in children and adolescents.

## Author contributions

JZ, WZ, and QG contributed to the conception of the study. JX and FC contributed significantly to analysis and manuscript preparation. JX, FC, DL, and XMS performed the data analyses and wrote the manuscript. XLS, ZP, TW, and QG contributed to the interpretation and discussion of the results of the analysis.

### Conflict of interest statement

The authors declare that the research was conducted in the absence of any commercial or financial relationships that could be construed as a potential conflict of interest.
